# Incidence and risk factors of acute kidney injury after femoral neck fracture in elderly patients: a retrospective case-control study

**DOI:** 10.1186/s12891-021-04966-3

**Published:** 2022-01-03

**Authors:** Sizheng Zhan, Wenyong Xie, Ming Yang, Dianying Zhang, Baoguo Jiang

**Affiliations:** 1 Department of Orthopedics, Peking University People’s Hospital, No.11 Xizhimen South Street, Xicheng District, Beijing, 100044 China; 2 Ministry of Education Key Laboratory of trauma treatment and nerve regeneration, Peking University People’s Hospital, Beijing, 100044 China; 3 Department of Orthopedics, Peking University Binhai Hospital, Tianjin, 300450 China

**Keywords:** Acute kidney injury, Femoral neck fractures, Risk factors

## Abstract

**Background:**

Hip fracture is highly associated with disability and consequently, mortality in the elderly population. Postoperative acute kidney injury (AKI) is not unusual and is associated with considerable morbidity and mortality. We aimed to determine the incidences and potential risk factors for postoperative AKI in elderly patients with femoral neck fracture.

**Methods:**

We retrospectively evaluated patients over 65 years of age who had been subjected to surgery for femoral neck fracture at Peking University People's Hospital from January 2015 to December 2019. Demographic characteristics and potential risk factors were collected. AKI was defined according to the Kidney Disease Improving Global Outcomes Guidelines (KDIGO).

**Results:**

A total of 308 elderly patients with femoral neck fracture were included in the study. The overall incidence of postoperative AKI was 12% (37 cases). Through binary logistic regression analysis, adjusted for age, intraoperative blood loss and BMI, we identified that early postoperative albumin levels, hemoglobin changes and intraoperative hypotension are independent risk factors for postoperative AKI. The model considering the three factors can improve accuracy of predicting the possibility of developing AKI. The patients with AKI had a significantly higher mortality of 40.5% than those without AKI (24.0%, *p* < 0.001)

**Conclusion:**

The incidence of postoperative AKI in elderly patients with femoral neck fracture was 12%. Independent risk factors for postoperative AKI included hemoglobin changes, early postoperative hypoalbuminemia and intraoperative hypotension. At the same time, postoperative AKI significantly increased mortality in elderly patients with femoral neck fracture. Taking multiple possible factors into consideration can better predict the possibility of elderly patients developing AKI after surgery.

## Introduction

Hip fracture is highly associated with disability and consequently, mortality [1, 2]. Although age-standardized annual incidences of hip fracture are gradually decreasing in many countries, they are high in the aging population [^1^]. Various studies have reported hip fracture-associated mortalities; however, their mechanisms have not been elucidated [3, 4].

Acute kidney injury (AKI) refers to the rapid deterioration (hours to days) of renal functions. Perioperative AKI is not unusual and is associated with considerable morbidity and mortality [5]. Surgical therapy itself, especially emergency and major surgery among critically ill patients, is associated with a high incidence of AKI [6].

Several studies on AKI following hip fracture have evaluated the incidences and risk factors for postoperative AKI [6–11]. Multiple studies [6, 10] have also reported that AKI following surgery in elderly hip fracture patients is associated with increased mortality after 3 months or 1 year. Common types of hip fractures include femoral neck fractures and intertrochanteric fractures, with femoral neck fractures being the most common. The two types of fractures exhibit significant differences in their mechanisms of fracture injury, surgical methods and postoperative rehabilitation [12, 13]. Therefore, it is necessary to clinically distinguish between the two to guide therapy.

This study aimed to determine postoperative AKI incidences following femoral neck fractures and the possible predictive factors for AKI in Beijing, China.

## Materials and methods

### Population

We retrospectively evaluated patients over 65 years of age who had been subjected to surgical therapy for femoral neck fractures at Peking University People's Hospital from January 2015 to December 2019. Prior to the start of the study, the protocol was approved by the Ethics Review Committee of Peking University People’s Hospital. The inclusion criteria for this study were as follows: i. Patients with femoral neck fractures without other combined fractures; ii. Patients aged over 65 years, irrespective of sex; iii. Patients subjected to surgical therapy involving total hip arthroplasty (THA), hemi-hip arthroplasty and internal fixation; and iv. Patients whose detailed and comprehensive medical records were available. The exclusion criteria for this study were as follows: i. A history of routine dialysis; ii. A history of mental illness; iii. Long-term use of nephrotoxic drugs; iv. Severe preoperative infection complications; and v. Unstable vital signs with death occurring soon after surgery (within 3 days).

### Date collection

#### Demographic characteristics

Standard clinical demographics that were collected included age, sex, body mass index (BMI), the period between the injury date and surgical date, and comorbidities such as dementia, hypertension, diabetes mellitus, pre-existing cardiovascular diseases, chronic obstructive pulmonary disease and chronic kidney disease.

#### Perioperative management

Serum hemoglobin, albumin and creatinine levels were preoperatively determined. The anesthesia method, surgical method, surgical time, intraoperative blood loss and intraoperative hemodynamic changes were recorded. Intraoperative hypotension was defined as a systolic blood pressure <80 mmHg or a mean blood pressure <55–60 mmHg lasting for 5 min [14]. Hemoglobin, albumin and serum creatinine (SCr) levels were measured on the first day after surgery and periodically during the first 7-day postoperative period. Postoperative blood transfusion history and postoperative complications were also recorded. The early postoperative albumin level was defined as the lowest serum albumin level on the first day after surgery. The hemoglobin change was defined as the difference between the preoperative hemoglobin level and the lowest hemoglobin level within seven days postoperatively, with or without blood transfusion.

#### Definition of AKI

The latest diagnostic criteria for AKI are included in the Kidney Disease Improving Global Guidelines (KDIGO) Clinical Practice Guidelines for Acute Kidney Injury of 2012 [15]. Patients were diagnosed with AKI if any one of the following conditions were met: an increase in serum creatinine levels by ≥0.3 mg/dl (≥26.5 µmol/L) within 48 h; an increase in serum creatinine levels by ≥1.5 times the baseline level within the previous 7 days; and urine volume ≤0.5 ml/kg/h for 6 h (Table 1). We selected changes in serum creatinine levels as the diagnostic criteria because only serum creatinine levels could be obtained retrospectively.

**Table 1:** Demographics of patients

	Overall *N*=308	With AKI *N*=37	Without AKI *N*=271	*P*-Valve
Age (mean ± SD)				0.052
	79.06±7.30	81.24±7.32	78.76±7.26	
Gender n (%)				1.000
Female	216 (70.1%)	26 (70.3%)	190 (70.1%)	
Male	92 (29.9%)	11 (29.7%)	81 (29.9%)	
Complications n (%)				0.953
Dementia	15 (4.9%)	3 (8.1%)	12 (4.4%)	
Hypertension	171 (55.5%)	20 (54.1%)	151 (55.7%)	
Diabetes mellitus	83 (26.9%)	10 (27.0%)	73 (27.0%)	
CHD	60 (19.5%)	8 (21.6%)	52 (19.2%)	
CVA	62 (20.1%)	8 (21.6%)	54 (20.0%)	
COPD	7 (2.3%)	1 (2.7%)	6 (2.2%)	
CRF	9 (2.9%)	1 (2.7%)	8 (3.0%)	
BMI (mean ± SD)				0.820
	24.10+3.31	24.09+3.32	24.22+3.31	
Time to surgery (mean ± SD)				0.842
	7.29+5.98	7.11+6.00	7.32+5.99	

*CHD* coronary heart disease, *CVA* cerebrovascular accident, *COPD* chronic obstructive pulmonary disease, *CRF* chronic renal insufficiency, *BMI* body mass index

#### Statistical analysis

All analyses were performed using IBM SPSS Statistics for Windows, version 26.0 (IBMCorp., Armonk, N.Y., USA). Between-group comparisons of categorical variables were performed by the chi-square test and Fisher’s exact test, whereas between-group comparisons of continuous variables were performed by the t-test. After adjusting for confounding variables, a generalized binary logistic regression analysis was performed. The alpha level was set to be *p* ≤ 0.05. SPSS 26.0 and STATA 15.0 were used to draw the ROC curves.

## Results

### Patient characteristics

Comparisons of the demographic characteristics between the AKI and non-AKI groups are shown in Table 1. A total of 308 elderly patients with femoral neck fractures, including 216 (70.1%) females and 92 (29.9%) males with a mean age of 79.06±7.3 years, were recruited in this study. The overall incidence of postoperative AKI was 12% (37 cases). There were no significant differences in age or sex between the two groups, nor were there differences in other characteristics, such as preoperative comorbidities, BMI or surgical time.

### Potential risk factors

The potential risk factors for postoperative AKI are presented in Table 2. The early postoperative level of serum albumin, hemoglobin changes and intraoperative hypotension were identified as significant risk factors for AKI (*p* < 0.01). Compared to the non-AKI group, the AKI group exhibited more intraoperative blood loss (*p*=0.036). There were no significant differences in preoperative creatinine levels, preoperative albumin levels, preoperative hemoglobin levels, preoperative mean arterial pressure (MAP), anesthesia methods, surgical methods, surgical time, blood transfusion history or postoperative complications between the two groups. After adjusting for age, intraoperative blood loss and BMI, early postoperative albumin levels, hemoglobin changes and intraoperative hypotension were found to be independent risk factors for postoperative AKI (Table 3).

**Table 2:** Potential risk factors

	Overall *N*=308	With AKI *N*=37	Without AKI *N*=271	*P*-Valve
Preoperative creatinine (umol/L)				0.643
	76.66+41.28	73.70+29.46	77.07+42.67	
Preoperative albumin (g/L)				0.402
	36.43+4.17	35.89+4.81	36.50+4.07	
Postoperative albumin (g/L)				0.000
	31.64+3.90	27.54+2.60	32.21+3.71	
Preoperative hemoglobin (g/L)				0.193
	115.42+17.68	113.72+11.98	117.12+19.22	
Hemoglobin change (g/L)				0.000
	8.6+11.73	22.3+15.85	6.73+9.68	
Preoperative MAP (mmHg)				0.352
	90.52+15.88	88.50+14.56	92.54+17.11	
Intraoperative hypotension				0.000
	25 (8.1%)	13 (35.1%)	12 (4.4%)	
Anesthesia				1.000
General anesthesia	71 (23.1%)	8 (21.6%)	63 (23.2%)	
Lumbar anesthesia	237 (76.9%)	29 (78.4%)	208 (76.8%)	
Surgery				0.681
Internal fixation	52 (16.9%)	8 (21.6%)	44 (16.2%)	
Hemi-hip arthroplasty	204 (66.2%)	24 (64.9%)	180 (66.4%)	
THA	52 (16.9%)	5 (13.5%)	47 (17.3%)	
Operation time (min)				0.163
	91.96+36.05	99.73+27.59	90.90+36.97	
Intraoperative blood loss (ml)				0.036
	126.24+126.58	167.03+152.24	120.65+121.92	
Blood transfusion				0.169
	181 (59%)	25 (67.6%)	156 (57.8%)	
Postoperative complications				0.353
Infection	32 (10.4%)	4 (10.8%)	28 (10.3%)	
Cerebral infarction	1 (3.2%)	1 (2.7%)	0 (0.0%)	
Pulmonary embolism	1 (3.2%)	0 (0.0%)	1 (2.7%)	
Delirium	1 (3.2%)	0 (0.0%)	1 (2.7%)	
Poor wound healing	1 (3.2%)	0 (0.0%)	1 (2.7%)	

*MAP* mean arterial pressure, *THA* total hip arthroplasty

**Table 3:** Binary logistic regression analysis, adjusted for age and BMI

AKI vs no-AKI	Beta	Odds Ratio	*P*-value
Postoperative albumin	-0.463	0.629	0.000
Hemoglobin change	0.128	1.137	0.000
Intraoperative hypotension	2.609	13.59	0.001
Blood loss	0	1	0.801
Age	0.049	1.05	0.219
BMI	0.106	1.112	0.190
Constant	3.207	-	-

### ROC curves for the significant risk factors

**Fig. 1:**
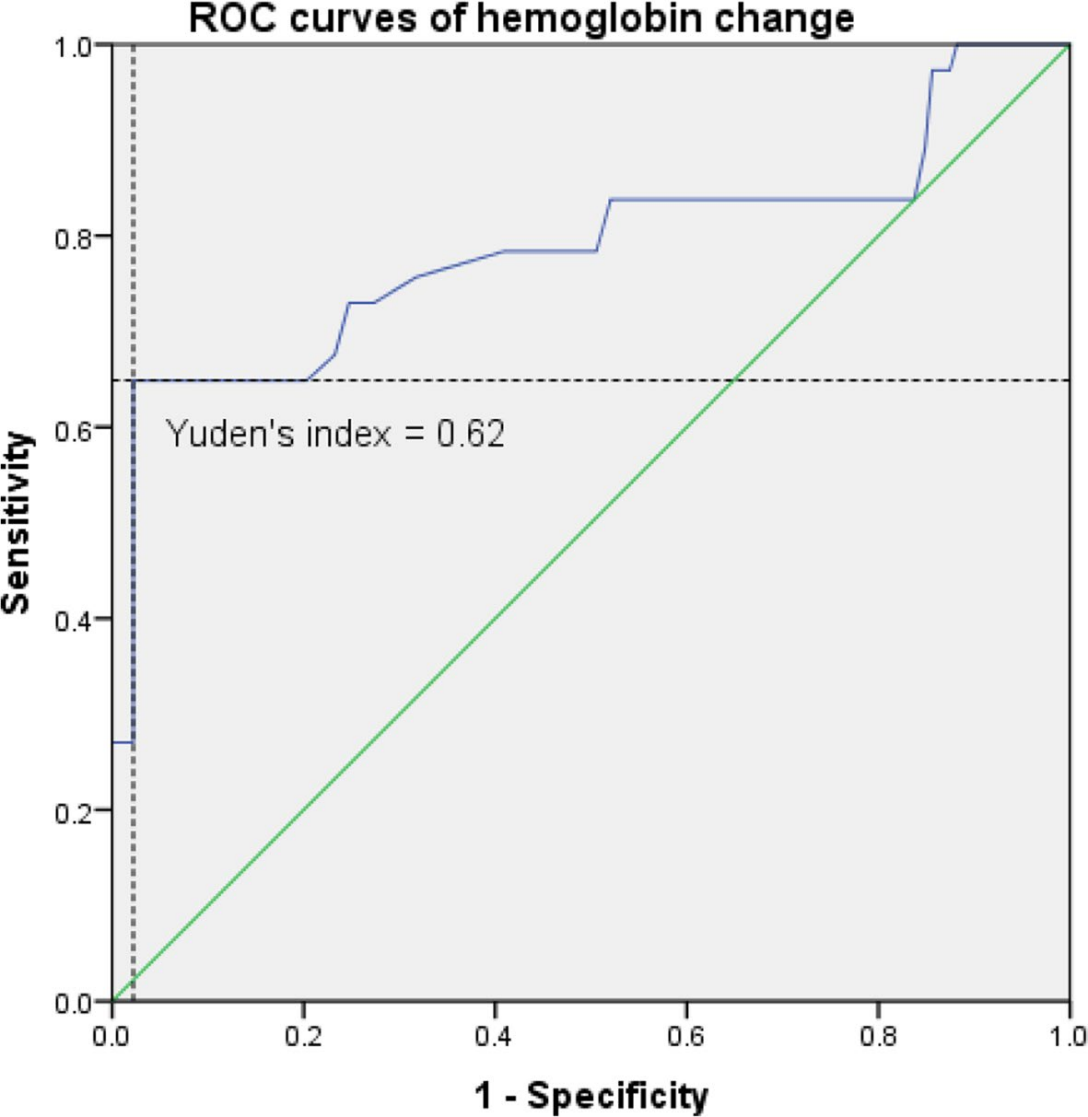
ROC curves of hemoglobin change predicting AKI. AUG: 0.789; Yuden’s index: 0.627; The cutoff value of the hemoglobin change was > 22.5 g/L; Sensitivity: 64.9%; Specificity: 97.8%

**Fig. 2:**
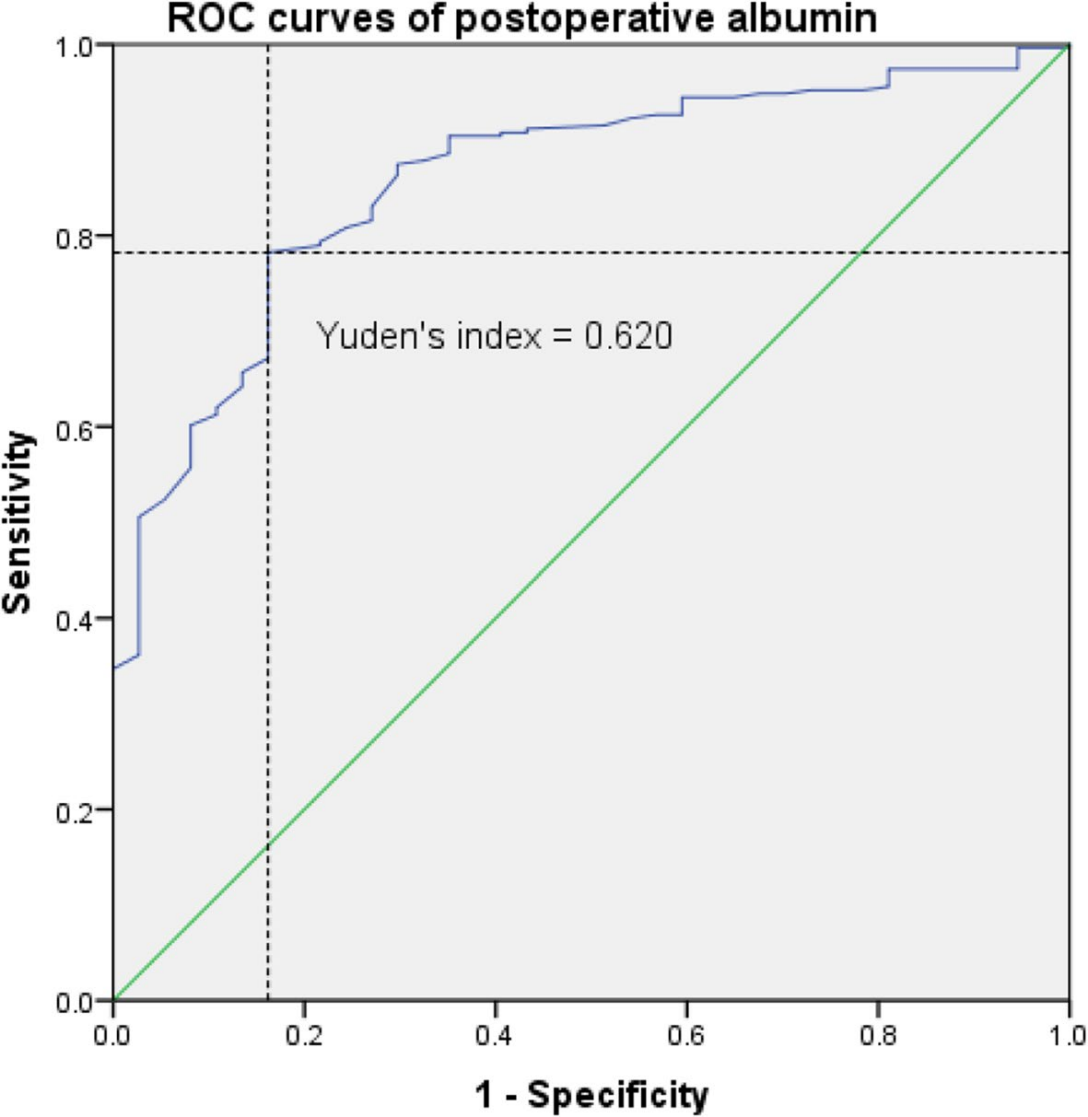
ROC curves of postoperative albumin levels predicting AKI. AUG: 0.859; Yuden’s index: 0.62; The cutoff value of the postoperative albumin was <29.6 g/L; Sensitivity: 78.2%; Specificity: 83.8%

**Fig. 3:**
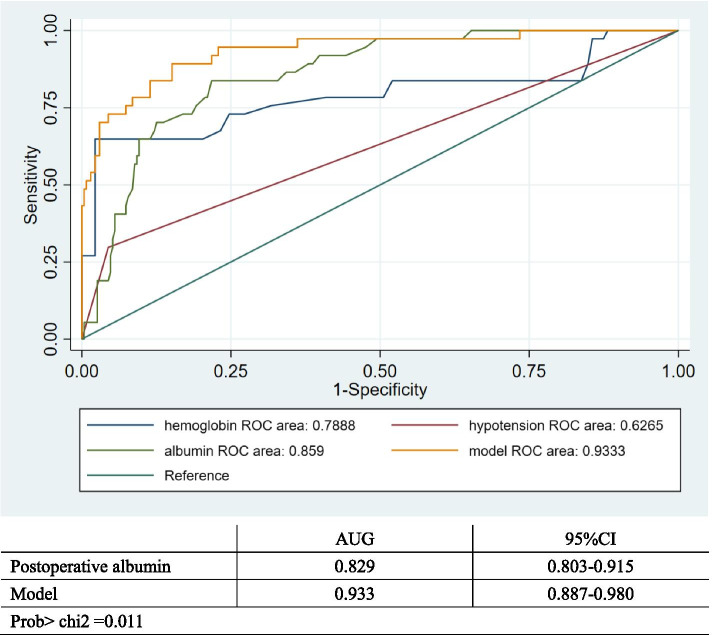
The ROC curves of the model and postoperative albumin, hemoglobin change and intraoperative hypotension

Figure 1 shows the ROC curves of hemoglobin changes predicting AKI. The area under the curve (AUC) was 0.789. The cutoff value of the hemoglobin change was > 22.5 g/L with a sensitivity of 64.9% and a specificity of 97.8%. Fig. 2 shows the ROC curves of postoperative albumin levels predicting AKI. The area under the curve (AUC) was 0.859. The cutoff value of postoperative albumin levels was <29.6 g/L with a sensitivity of 78.2% and a specificity of 83.8%. Based on the beta factors of the previous binary logistic regression, we proposed a formula for predicting the risk of postoperative AKI: model=3.207 + 0.128* hemoglobin change (g/L) -0.463* postoperative albumin level (g/L) +2.609* intraoperative hypotension (Yes, 1; No, 0). The ROC curves of the model and the other three independent risk factors are shown in Fig. 3. The model was significantly superior to the postoperative albumin level (*p*=0.011) and other independent risk factors.

### 1-year cumulative mortality

**Fig. 4:**
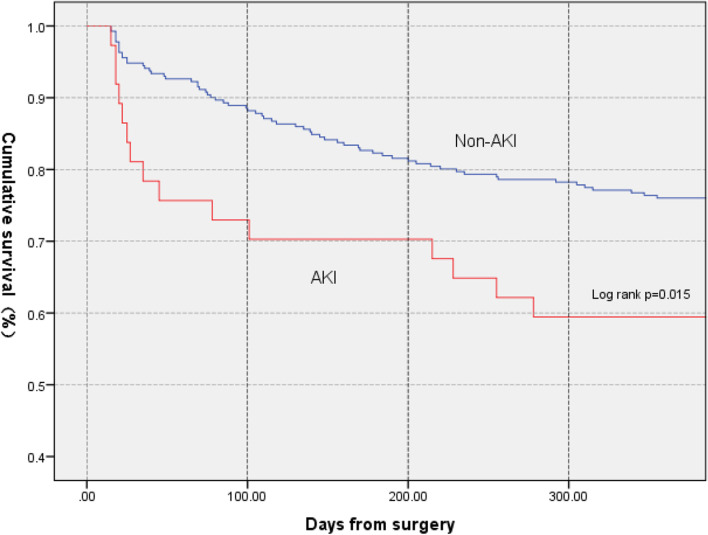
1-year survival for patients undergoing surgery for femoral neck fracture with and without AKI

At the 1-year follow-up, the overall cumulative mortality was 26.0%. The patients with any AKI had a significantly higher mortality of 40.5% than those without AKI (24.0%, *p *< 0.001, Fig. 4). Most of these deaths occurred within three months after surgery.

## Discussion

In our study population, the incidence of postoperative AKI was 12%, slightly lower than the 11.8%-28.4% reported by previous studies for hip fracture patients. These differences can be attributed to various factors, such as the varied definitions of AKI, varied monitoring periods, and heterogeneity of the selected patients. However, we do agree that elderly patients with hip fractures or femoral neck fractures are at a greater risk of postsurgical AKI. Compared with previous studies, we focused only on patients with femoral neck fractures, rather than all types of hip fractures.

Shin et al.'s findings [7–9, 16] suggested that postoperative hypoalbuminemia, substantial blood loss, and intraoperative hypotension are associated with postoperative AKI in patients with hip fracture. The conclusions of our study were consistent with previous studies to some extent, but we found no significant differences in preoperative albumin levels, preoperative hemoglobin levels, preoperative MAP or other baselines between the two groups. Therefore, we can boldly conclude that the occurrence of postoperative AKI in patients with femoral neck fracture is mainly related to intraoperative and postoperative blood flow hypoperfusion. Of course, there are maybe other possible reasons for postoperative AKI caused by hypoalbuminemia and anemia^9^. Therefore, we recommend timely treatment for patients with postoperative albumin levels lower than 29.6 g/L or postoperative hemoglobin decreases greater than 22.5 g/L.

Some studies [17–19] have reported that blood transfusion is also a risk factor for postoperative AKI in hip fracture patients. As a result, we seem to be caught in a paradoxical dilemma. The cause of AKI caused by blood transfusion is not clear. Some studies suggest that it may be caused by immunological responses induced by blood transfusions. Therefore, we are more inclined to believe that the association between blood transfusions and AKI may be the expression of the patient's primary disease, such as anemia and hypotension, rather than the blood transfusion itself causing AKI. Therefore, we recommend blood transfusion for patients who meet transfusion indications.

Many other risk factors for AKI after hip surgery, such as age, preoperative albumin levels, BMI, preoperative complications, and postoperative complications, have been reported [18, 20, 21]. In this study, these indicators were not found to be significantly different between the AKI and no-AKI groups. These outcomes could be attributed to differences in the populations for different studies. We cannot deny that these factors are high-risk factors for postoperative AKI in patients with femoral neck fracture. Therefore, they should be taken into account in clinical work to prevent AKI. More prospective large sample studies are needed to confirm these possible risk factors.

The proposed prediction formula was confirmed by the ROC curve. Its predictive ability was superior to that of hemoglobin changes, early postoperative hypoalbuminemia and intraoperative hypotension alone. The AUG of the model was significantly larger than the AUG of postoperative albumin levels and hemoglobin changes, indicating that the model could find the optimal solution to balance sensitivity and specificity in predicting postoperative AKI. Using this formula, we intend to demonstrate that multiple factors should be integrated and analyzed to evaluate patients' risk for postoperative AKI.

By clarifying the risk factors for postoperative AKI in patients with femoral neck fracture, clinical work can be better guided to avoid the occurrence of postoperative AKI. Good intraoperative blood pressure control and the timely correction of hypoalbuminemia and anemia during the perioperative period can effectively prevent the occurrence of AKI. At the same time, we can use more advanced and accurate markers for the early diagnosis of AKI in patients to achieve early treatment, such as retinol-binding protein, neutrophil gelatinase associated lipocalin and cystatin C which have been proven sensitive for the early detection of AKI [22, 23]. For some patients with high-risk factors for AKI, we should pay more attention to the regular test results of patients, and if necessary, use preventive drugs for AKI.

The limitations of this study are as follows. First, it was a retrospective study; therefore, it is necessary to perform a large-scale prospective study to confirm our findings. Second, since this was a retrospective study, the long-term follow-up of patients was not possible to observe changes in long-term creatinine levels and patient survival. Third, due to the limited sample size, staging and related studies of postoperative AKI were not performed. Fourth, sCr levels can be influenced by volume overload, nutrition, steroids, and muscle trauma [24]. The immediate postoperative period sCr concentrations can be lower than at baseline as a result of hemodilution after massive fluid administration and fluid shifts. Biomarkers for the early detection of AKI [22, 23] should be evaluated in future studies.

## Conclusion

The incidence of postoperative AKI in elderly patients with femoral neck fracture was 12%. Independent risk factors for postoperative AKI included perioperative hemoglobin changes, early postoperative hypoalbuminemia and intraoperative hypotension. At the same time, postoperative AKI significantly increased mortality in elderly patients with femoral neck fracture. Taking multiple possible factors into consideration can better predict the possibility of elderly patients developing AKI after surgery.

## Data Availability

The datasets generated and analyzed during the current study are not publicly available due to the data also forms part of an ongoing study but are available from the corresponding author on reasonable request.

## Abbreviations

AKI: acute kidney injury
THA: total hip arthroplasty
BMI: body mass index
SCr: serum creatinine
KDIGO: Kidney Disease Improving Global Guidelines
AUC: The areas under curve
MAP: mean arterial pressure
